# A novel record of African wild dogs (*Lycaon pictus*) in Vwaza Marsh Wildlife Reserve, Malawi

**DOI:** 10.1002/ece3.10671

**Published:** 2023-10-31

**Authors:** Olivia Sievert, Matthias Hammer, Eleanor Comley, Benjamin Hintz, William O. Mgoola, Robert S. Davis

**Affiliations:** ^1^ Lilongwe Wildlife Trust Lilongwe Malawi; ^2^ Endangered Wildlife Trust Midrand South Africa; ^3^ Biosphere Expeditions Dublin Ireland; ^4^ Department of National Parks and Wildlife Malawi Lilongwe Malawi; ^5^ Department of Conservation Management Nelson Mandela University George Western Cape South Africa

**Keywords:** camera traps, dispersal, large carnivores, Malawi, Zambia

## Abstract

Most African wild dog (*Lycaon pictus*) populations are in decline and, due to habitat fragmentation and conflict rates in areas of higher anthropogenic land‐use, are primarily restricted to protected areas. As a species that occurs at low densities, with a strict reproductive social structure, wild dogs rely on long‐range dispersal to facilitate colonization, reproduction, and pack formation. In Malawi, large carnivores have been subject to widespread population decline and several protected areas have a reduced large carnivore guild, including the loss of resident wild dog populations. Here, during a biodiversity monitoring camera trap survey, we captured a novel record of wild dogs in Vwaza Marsh Wildlife Reserve (Vwaza), Malawi. The 11 photographic captures of potentially three individual wild dogs represent the first documented evidence of the species in Vwaza and the first record since an unconfirmed report in 2011. We hypothesize that this group of wild dogs moved into Vwaza through the Malawi‐Zambia Transfrontier Conservation Area (MZTFCA), with the MZTFCA linking protected areas in Malawi with the Luangwa Valley, Zambia. The evidence provided here, and similar documentation in Kasungu National Park, Malawi, show that large carnivores can potentially move through the MZTFCA into protected areas in Malawi. We argue that the MZTFCA provides an important dispersal corridor that could help facilitate the recolonization of wild dogs, and other large carnivores, in Malawian protected areas. However, further research is needed to assess the permeability and status of the MZTFCA corridor into Malawi. We show that camera traps can be useful to document novel records of rare species and can be used to inform conservation management planning.

## Graphical abstract

Malawi's large carnivore populations have been subject to widespread population decline, including the extirpation resident African wild dogs. Here, we report on findings from a biodiversity monitoring survey, where we captured a novel record of African wild dogs in Vwaza Marsh Wildlife Reserve, Malawi. These photographs represent the first documented evidence of African wild dogs in Vwaza Marsh Wildlife Reserve and demonstrate connectivity within the northern parts of the Malawi‐Zambia Transfrontier Conservation Area.

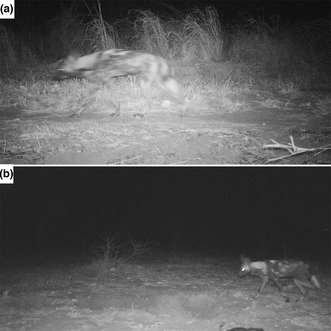

## 1 INTRODUCTION

The African wild dog (*Lycaon pictus*; hereafter wild dog) is classified as endangered, with approximately 1400 breeding individuals remaining across 39 subpopulations (Woodroffe & Sillero‐Zubiri, 2020). Once widespread across Sub‐Saharan Africa, wild dogs have lost approximately 93.2% of their historical range (Wolf & Ripple, 2017) with habitat fragmentation, human persecution and infectious disease as the primary drivers of their continued population decline (Woodroffe & Sillero‐Zubiri, 2020). As a result, viable wild dog populations are largely restricted to protected areas (Cozzi et al., 2020; Woodroffe & Sillero‐Zubiri, 2020). The relative isolation of wild dog subpopulations, combined with their low population densities and strict reproductive social structure, means the species is particularly reliant on dispersal for colonization, pack formation, and reproduction (Cozzi et al., 2020; Woodroffe et al., 2020). Furthermore, their large spatial requirements, especially during the dispersal stage, mean they often stray beyond protected area boundaries and into conflict with humans (van der Meer et al., 2014; Woodroffe et al., 2007).

Malawi, in south‐central Africa, has undergone a significant decline in large carnivore populations (Davis et al., 2021; Mésochina et al., 2010; Munthali & Mkanda, 2002). Severe under‐funding for conservation and park management, combined with increasing anthropogenic pressures around park boundaries, has resulted in protected areas being subject to high levels of poaching and habitat destruction (Munthali & Mkanda, 2002; van Velden et al., 2020). Particularly for wide‐ranging, social, and less‐cryptic species, such as wild dog and lion (*Panthera leo*), the loss of abundant prey bases and edge effects surrounding protected areas have led to near‐extirpation within Malawi (Davis et al., 2021; Mésochina et al., 2010; Purchase et al., 2007). Although some parks under public–private partnerships have begun to recover large carnivore populations through translocation and reintroduction projects (e.g., Briers‐Louw et al., 2019; Sievert, Fattebert, et al., 2022), most of Malawi's protected areas are reliant on corridor protection, reserve restoration, and natural recolonization to increase large carnivore populations. As several protected areas in Malawi have transboundary initiatives with the Luangwa Valley system in Zambia, they are optimal areas for dispersal and potential recolonization (Davis et al., 2021; Sievert, Evans, et al., 2022).

Here, using data from a recent camera trap survey, we document a novel record of a group of wild dogs in Vwaza Marsh Wildlife Reserve (hereafter Vwaza), Malawi. Although considered part of the current wild dog distribution range (Woodroffe & Sillero‐Zubiri, 2020), evidence of the species' status in Vwaza is extremely limited, with the last unconfirmed report in 2011 (Department of National Parks and Wildlife, 2011). As such, these images represent the first photographic evidence of wild dogs in Vwaza. We discuss the significance of this result for large carnivore recolonization in Malawi and the potential implications for connectivity and dispersal corridors across the wider Malawi‐Zambia Transfrontier Conservation Area (MZTFCA).

## 2 METHODS

### 2.1 Vwaza Marsh Wildlife Reserve and Malawi‐Zambia Transfrontier Conservation Area

**FIGURE 1:**
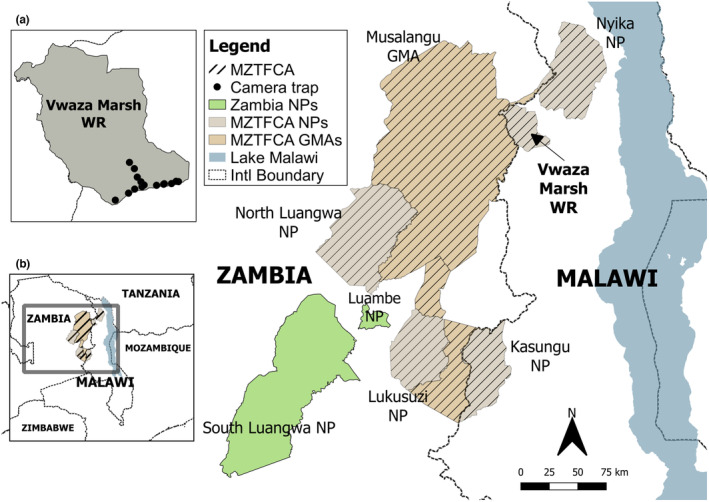
Map showing the location of Vwaza Marsh Wildlife Reserve (Vwaza) and its position within the wider Malawi Zambia Transfrontier Conservation Area (MZTFCA). Inset (a) displays the location of camera traps deployed in Vwaza during the survey and (b) displays the location of the MZTFCA in south‐central Africa, with the MZTFCA highlighted.

This study was conducted in Vwaza Marsh Wildlife Reserve, Malawi (33°28′ E, 11°00′ S; 986 km^2^). The reserve consists primarily of miombo woodland (*Brachystegia* spp.), interspersed with wetland areas that are seasonally flooded by the Luwewe and South Rukuru rivers (Engel et al., 2013; Mgoola & Msiska, 2017). Vwaza forms part of the 32,278 km^2^ MZTFCA (Figure 1), a significant area for biodiversity conservation in the Central Zambezian Miombo Woodland Ecoregion that was established in 2003 (Peace Parks Foundation, 2023). The MZTFCA consists of Vwaza, Kasungu National Park and Nyika National Park in Malawi and Lukusuzi National Park, North Luangwa National Park and several game management and forest reserve areas in Zambia. The MZTFCA is split into two main components, with Vwaza, which borders Lundazi Forest Reserve in Zambia, forming part of the Nyika‐North Luangwa TFCA.

### 2.2 Camera trap surveys

A total of 17 infrared camera traps (16 Bushnell Trophy Cam; Bushnell Corporation; 1 Reconyx HyperFire; Reconyx) were deployed across the southern section of Vwaza (Figure 1a), with an average trap spacing of 1.38 km (SD ± 0.96). Three camera traps were baited with goat carcasses to increase carnivore detection rates, as one of the key objectives of the survey was to provide an inventory of carnivores in the park. Camera placement focused on the southern section of the park due to the limited road network in the north. As large carnivores were a key focus of the biodiversity survey, camera traps were primarily placed along the Vwaza road network to maximize detection rates (Davis et al., 2021). Camera traps were attached to trees ~80 cm above the ground and facing the road. Camera traps were programmed with the same settings (16MP image size, 10 s interval, medium sensitivity, medium night vision shutter speed), and took two images consecutively, allowing for more reliable captures of multiple individuals in herds or groups. Camera traps were originally set up during a Biosphere Expedition citizen science biodiversity monitoring project (September 25, 2022 – October 07, 2022), with the 14 non‐baited cameras left in place until October 22, 2022. All pictures were analyzed using Timelapse2 (v2.3.0.0; Greenberg, 2019). Images of individual wild dogs were identified to the individual‐level where possible, using unique pelage patterns and timestamp information.

## 3 RESULTS

**FIGURE 2:**
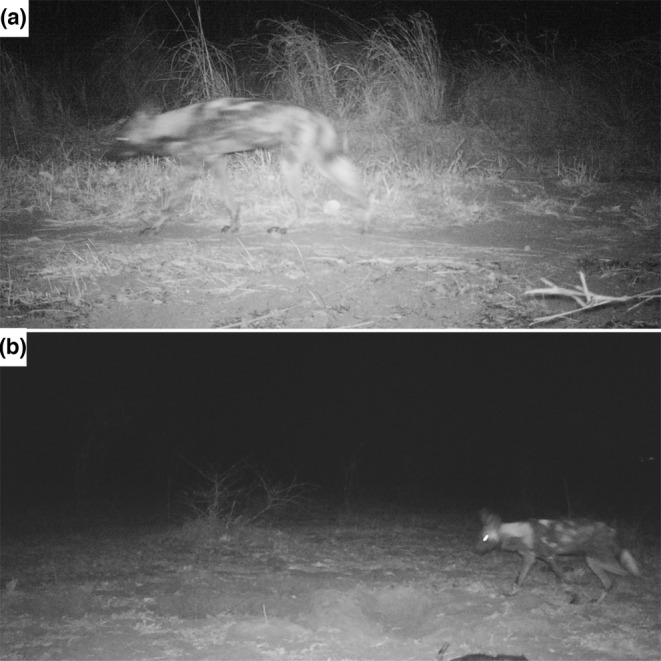
Camera trap images of left flank pelage patterns that identify two individual African wild dogs in Vwaza Marsh Wildlife Reserve, Malawi. Panel a is from September 24, 2022, the first documented images of the species in the reserve. Panel b is from October 04, 2022 at a different camera trap station.

**FIGURE 3:**
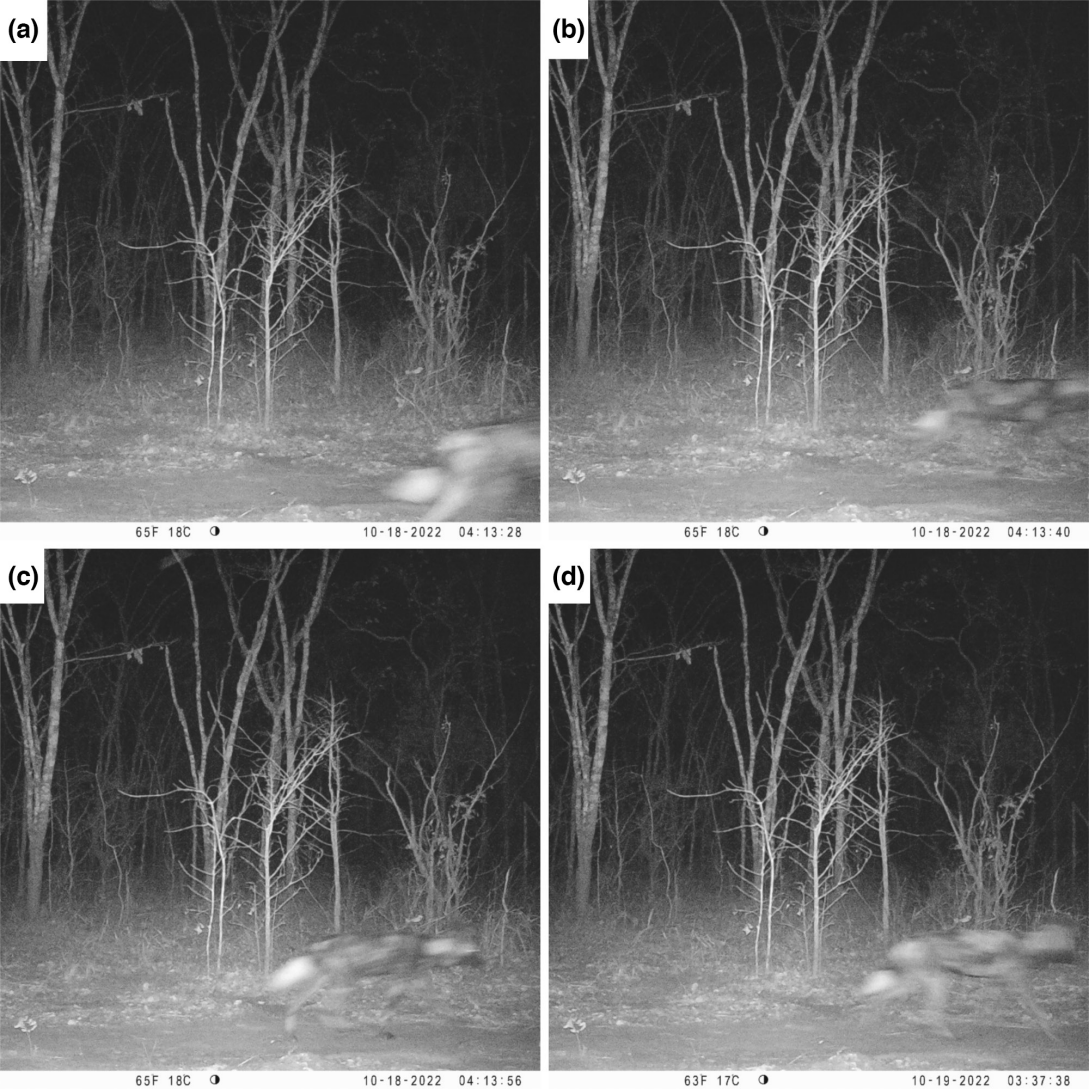
Four camera trap images of African wild dogs at the same camera trap station in Vwaza Marsh Wildlife Reserve, Malawi. (a) The first recorded image of a wild dog (18 October 2022); (b) the following image and from the time stamp and the difference in pelage patterns, we believe this is likely to be a second individual; (c) a different individual from b, based on pelage patterns and the time stamp; (d) an image from the same camera trap station (October 19, 2022); it is difficult to say with certainty if this is a new individual based on right‐flank pelage patterns. From these images, we suggest that there were likely three individual wild dogs in Vwaza during the camera trap survey.

During the survey, 11 images of wild dogs were obtained from four different observation events at three camera stations. Individual identification from pelage patterns and estimates of the number of wild dogs observed in Vwaza were limited by the quality of infrared images. From left flank images, we could identify two different individuals (Figure 2). Right flank images were harder to identify (Figure 3); however, from the series of timestamps on three photographic captures there was evidence that potentially three different wild dogs moved past the camera trap in quick succession. Therefore, we are confident there were a minimum of two individual wild dogs, with a third individual likely based on timestamps and potential variation in pelage patterns. All photographic captures were recorded at night between 06:47 p.m. and 04:13 a.m.

## 4 DISCUSSION

Through our camera trap survey, we have confirmed the first photographic records of wild dogs in Vwaza and provided evidence that wild dogs are still present, though likely transient, in the protected area. Our findings support Davis et al. (2021), who reported the presence of dispersing wild dogs and lions in Kasungu National Park (KNP), Malawi, and attributed this to dispersal corridors through the MZTFCA. Encouragingly, we documented a group of at least two wild dogs in Vwaza, whereas the same single wild dog, confirmed through pelage patterns, was recorded in KNP during camera trap surveys in 2017 and 2018 (Davis et al., 2021). As camera trap surveys have previously taken place in Vwaza in 2018 and 2019 (see Harwood et al., 2019, 2020), and there have been no observations from park management since the time of this survey (Matthews Mumba, personal communication), it is unlikely the group is resident. It is therefore assumed that this was either a dispersal group containing cohorts of a single sex or a splinter‐group from the Zambian‐side of the MZTFCA. We feel this conclusion is reasonable, because of the proximity of the Luangwa Valley and this region holding the largest wild dog population in Zambia (Creel et al., 2020). Our wild dog images from Vwaza were cross‐referenced with a wild dog database maintained by Zambia Carnivore Program that encompasses parts of the MZTFCA, but could not be linked to known individuals in the database (Matthew Becker, personal communication). However, as this database is missing individuals that reside outside of the core protected areas and acknowledging the low quality of the images provided for identification, it is still probable that these wild dogs moved into Vwaza from the Zambian‐side of the MZTFCA.

Wild dogs require large areas of connected habitat and often exhibit long‐range dispersal events, with such movements promoting source‐sink dynamics by facilitating recolonization and supporting viable populations at national and international scales (Cozzi et al., 2020; Creel et al., 2020). As there was no evidence of wild dog pups in these images, toward the end of the denning season (May–September; Comley et al., 2023), and considering the size of the observed group, we believe it is likely the Vwaza images are of a dispersal group or splinter pack. Our evidence of a wild dog group in Vwaza, alongside similar findings in KNP (Davis et al., 2021), suggests that recolonization, or at least supplementation, of Malawi's protected areas from source populations in the MZTFCA, or within close proximity (Luambe NP, North Luangwa NP, South Luangwa NP), could be possible. We believe that management initiatives are needed to increase law enforcement and restore prey bases to abundant levels in these protected areas, as the issues of poaching and insufficient prey numbers will hinder dispersal and subsequent recolonization efforts (DNPW, 2011).

Anthropogenic land‐use, particularly the intensity of human activities, has been identified as the key factor influencing connectivity and landscape permeability for wild dogs (Cozzi et al., 2020; Creel et al., 2020; Hofmann et al., 2021). As the human footprint index increases around the MZTFCA (Creel et al., 2020; Watson et al., 2015), these pressures may hinder future dispersal attempts and further research is needed to assess connectivity and permeability between Malawian protected areas and potential dispersal corridors. The distance between potential source populations in Zambia (<300 km) and Vwaza is within the limits of known wild dog dispersal ranges, so establishing if the lack of recorded dispersal events is due to insufficient monitoring in Malawi's protected areas or barriers to dispersal along MZTFCA movement corridors is an essential step for conservation planning. As there have been unconfirmed wild dog reports in Malawian protected areas over the last few years, it is important that observations are properly reported, and that training is provided for correct identification and reporting standards. We encourage further reporting of novel dispersal events for threatened large carnivores and suggest that the MZTFCA could provide a valuable conservation tool for restoring large carnivores in Malawi's protected areas.

## AUTHOR CONTRIBUTIONS

**Olivia Sievert:** Conceptualization (equal); methodology (equal); project administration (equal); writing – original draft (supporting); writing – review and editing (equal). **Matthias Hammer:** Conceptualization (equal); data curation (supporting); methodology (equal); project administration (equal); writing – review and editing (supporting). **Eleanor Comley:** Data curation (supporting); writing – review and editing (equal). **Benjamin Hintz:** Conceptualization (equal); data curation (lead); methodology (equal); project administration (equal). **William O. Mgoola:** Writing – review and editing (equal). **Robert S. Davis:** Visualization (lead); writing – original draft (lead); writing – review and editing (equal).

## CONFLICT OF INTEREST STATEMENT

The authors declare no competing interests.

## Data Availability

Wild dog images are presented in the manuscript. No coding or additional analyses were conducted.
